# Consumer Behavior Toward Andean Grains Among Young Peruvian Adults: Purchase Patterns, Motivations, and Barriers Across Cultural Contexts

**DOI:** 10.3390/foods15183341

**Published:** 2026-09-20

**Authors:** Dany Yudet Millones-Liza, Elizabeth Emperatriz García-Salirrosas, Manuel Escobar-Farfán, Iván Veas-González, Manuel Amasifuen-Reategui, Esther Olivia Acosta-Miraval

**Affiliations:** 1 Escuela Profesional de Administración, Facultad de Ciencias Empresariales, Universidad Peruana Unión, Lima 15102, Peru; dannie@upeu.edu.pe; 2 Faculty of Management Science, Universidad Autónoma del Perú, Lima 15842, Peru; 3 Department of Administration, Faculty of Administration and Economics, University of Santiago of Chile, Santiago 9170020, Chile; manuel.escobar@usach.cl; 4 Departamento de Administración, Facultad de Economía y Administración, Universidad Católica del Norte, Antofagasta 1270709, Chile; iveas@ucn.cl; 5 Unidad de Ciencias Empresariales, Escuela de Posgrado, Universidad Peruana Unión, Lima 15102, Peru; manuel.amasifuen@upeu.edu.pe; 6 Universidad Tecnológica Del Perú, Lima 15487, Peru; c27178@utp.edu.pe

**Keywords:** Andean grains, quinoa, consumer behavior, purchase barriers, health motivations, cultural context, purchase frequency, Peru

## Abstract

Andean grains, such as quinoa (*Chenopodium quinoa* Willd.), kiwicha (*Amaranthus caudatus*), cañihua (*Chenopodium pallidicaule* Aellen), and tarwi (*Lupinus mutabilis* Sweet), represent a nutritional and cultural heritage of paramount importance to Peru; however, their regular consumption remains limited despite widespread recognition of their beneficial properties. This study analyzes consumer behavior toward Andean grains among Peruvian individuals, characterizing purchasing and consumption patterns, motivations, and barriers, and examining their associations with sociodemographic variables within the cultural context. We administered a structured cross-sectional survey to 1074 Peruvian consumers using non-probability convenience sampling. We used descriptive frequency analyses and chi-square tests with Cramer’s V to evaluate associations between sociodemographic and behavioral variables. Quinoa was the most consumed grain (79.3%), followed by kiwicha (47.0%), cañihua (31.1%), and tarwi (7.9%). Most purchases and consumption practices were occasional (47.0% and 42.6%, respectively), with raw grain for home cooking being the dominant presentation (47.3%) and the local market being the main purchasing channel (36.4%). The main motives for buying were the nutritional properties (63.6%) and the product’s natural character (46.2%), whereas cultural identity was a relevant secondary motive (21.8%). The most frequent barriers were high price (30.7%), lack of knowledge about preparation (26.9%), and lack of time (21.4%), which together accounted for 79.0% of the responses. Chi-square analyses revealed that cultural background showed the most consistent sociodemographic associations, with significant associations ranging from small to moderate (V = 0.099–0.169). Consumers from the traditional Andean context showed higher purchase frequency (37.3%) and greater use of alternative channels (26.1%) than those from the urban-coastal context, while in the latter, lack of time was the leading barrier (27.3%), tied with price. These findings reveal a pattern consistent with the gap between the positive valuation of these foods and their regular inclusion in the diet, and highlight the need for multidimensional interventions that address economic, cognitive, and time barriers to expand regular Andean grain consumption among Peruvian consumers, particularly in young urban segments.

## 1. Introduction

Andean grains like quinoa (*Chenopodium quinoa* Willd.), kiwicha (*Amaranthus caudatus*), cañihua (*Chenopodium pallidicaule* Aellen), and tarwi (*Lupinus mutabilis* Sweet) are a unique source of nutrition for Andean cultures and have been acknowledged as important foods by the United Nations Food and Agriculture Organization (FAO) due to their high protein content, essential amino acids, fiber, and adaptability to adverse climatic and soil conditions [1,2]. Increased interest in plant-based, functional, and healthy food products has positioned Andean grains as attractive ingredients that can help people to prevent chronic diseases [3,4]. Despite Peru being the largest producer and exporter of these products globally, their domestic consumption remains predominantly occasional and very low in the domestic market, thus creating a paradox between productive capacity and dietary incorporation and consumption of Andean crops [5,6].

Consumer behavior regarding Andean grains is shaped by an intricate interplay among purchasing and consumption modes, motivations, and barriers. According to the literature on consumer behavior regarding healthy foods, three key factors explain why consumers choose them: (1) Health-related motives and cognitive factors lead to healthy food purchasing [7]. (2) Sociodemographic and economic factors influence access to and purchase of foods, such that higher levels of income and education are usually associated with greater uptake of healthy foods [8,9]. (3) There are cultural and geographical determinants of food behavior, implying that various regions have different values, habits, and perceived barriers to the use of certain foods [10,11]. Nevertheless, these conclusions come from studies of food consumption in general and have not been tested on food groups of significant cultural and historical importance, such as Andean foods in Peru.

In Peru, this gap is particularly important. Peru has established itself as the world’s top producer and exporter of Andean grains, though local consumption remains low [5]. This situation refers to the gap between the favorable assessments of foods and food consumption practices in the healthy food literature [12,13]. Even though Andean grains are widely recognized for their nutritional and cultural value, a considerable proportion of Peruvians still do not use them regularly in their diet. To understand this trend, it is necessary to examine what consumers do, how often they consume them, how much they buy, which channels they use, what drives them, and why they consume Andean grains infrequently.

Research on Andean grain consumption in Peru has mainly focused on nutritional characteristics and international market issues [14,15,16,17] or tested explanatory theories for purchase intention in particular student groups [6]. Nevertheless, no comprehensive, statistically valid analysis of consumption patterns, motivations, and obstacles in Peru exists. Moreover, the role of sociodemographic factors, especially consumers’ cultural backgrounds, in this process remains underexplored. This gap limits opportunities to develop actionable interventions to improve consumption of Andean grains in Peru.

In light of this, this study addresses the following research question: what purchase and consumption patterns, motivations, and barriers characterize Peruvian consumers’ behavior toward Andean grains, and how do these differ by cultural context of origin, age group, and sex? To address this question, the study pursues four objectives: (1) to characterize the purchase and consumption patterns of Andean grains within Peruvian society, as well as what types of grains are usually consumed and how often these purchases are made; (2) to identify the main purchase reasons among the surveyed consumers behind their purchases of Andean grains; (3) to analyze the key barriers limiting regular Andean grain consumption among the participants; and (4) to examine the associations between sociodemographic variables, such as cultural background, age, and sex, and some behavioral characteristics of the Andean grain consumers.

As this is the first study to examine Andean grain consumers of this type, it will target the young consumer segment, university students and individuals aged 18–30, whose food habits and eating practices are important for improving understanding of future patterns of Andean grain consumption in Peru.

The findings of this study provide empirical evidence useful to three target groups: first, decision-makers and health professionals, because it provides a report on the key limitations to regular consumption and on the sociodemographic groups facing the most hurdles; second, stakeholders in the sphere of the food industry, as it pinpoints the motivational aspects associated with health and culture important for implementing marketing strategies; and third, researchers, as it establishes empirical associations between cultural context and behavioral outcomes that future explanatory studies can formally model.

## 2. Literature Review and Theoretical Background

### 2.1. Consumer Behavior Toward Healthy and Traditional Foods

Consumer behavior encompasses the cognitive, affective, and behavioral processes involved in food selection, purchase, and consumption, shaped simultaneously by individual, social, and contextual factors [18,19]. A well-documented phenomenon in this field is the gap between positive food evaluations and actual consumption behavior: consumers consistently report favorable attitudes toward healthy, natural, and sustainable foods, yet these dispositions do not reliably translate into regular purchasing or consumption patterns [13,20,21]. This gap is especially pronounced for functional and traditional foods, where price, limited culinary knowledge, poor availability, and time constraints suppress consumption even when attitudinal dispositions are favorable [22].

Research on the motivations driving healthy food consumption identifies health consciousness as a central antecedent of purchase intention [6,23,24]. Alongside health motivations, cultural identity plays a meaningful role in traditional food consumption: consumers associate these products with origin, custom, and cultural belonging, generating symbolic bonds that extend beyond nutritional content [25,26]. The coexistence of functional, symbolic, and habitual motivational dimensions is characteristic of traditional food categories and must be recognized in promotional strategies [21,27].

Barriers to healthy food consumption operate at economic, cognitive, temporal, and logistical levels [20]. Price has been consistently identified as the dominant barrier, with the strongest impact in low-income contexts [28]. Lack of culinary knowledge and time scarcity constitute cognitive and temporal barriers that prevent regular home preparation, particularly in urban environments [29,30]. Cultural and territorial contexts further shape consumer behavior, as regional differences influence values, habits, and perceived barriers to specific food categories [10,11].

### 2.2. Andean Grains: Nutritional Value, Market Context, and Research Gap

Andean grains present exceptional nutritional profiles: quinoa is characterized by high-quality protein (14–18 g per 100 g dry matter) with an essential amino acid profile comparable to casein; cañihua exceeds quinoa in protein content in several parameters and contains no bitter saponins; and tarwi offers one of the highest plant-source protein contents (32–52 g per 100 g dry matter), comparable to soybean [17,31,32]. Despite these attributes, consumption of cañihua and tarwi remains markedly lower than that of quinoa, reflecting asymmetries in commercial visibility and consumer familiarity rather than differences in nutritional quality [33].

Peru has consolidated its position as the world’s leading quinoa producer and exporter [34]. Yet this export success contrasts with predominantly occasional domestic consumption. The international success of quinoa has paradoxically contributed to rising domestic prices, reducing affordability for the local population, a phenomenon described as the quinoa paradox [14,15]. In terms of distribution, local markets and proximity channels retain a central role in Andean grain purchasing in Peru, alongside a smaller but growing segment of alternative channels such as organic fairs and direct-from-producer sales [35,36].

Prior research on Andean grain consumption has focused primarily on nutritional attributes and global market dynamics, or has tested explanatory models of purchase intention in specific student populations [6]. A simultaneous characterization of purchase and consumption patterns, motivations, and barriers among Peruvian consumers is lacking, as is a systematic examination of how these dimensions vary by cultural context of origin. This dual descriptive and inferential gap limits the development of targeted interventions to expand regular Andean grain consumption in Peru.

## 3. Materials and Methods

### 3.1. Participants

Because a comprehensive sampling frame of consumers of these products was not available, a non-probability convenience sampling method was used, a strategy frequently employed in exploratory and descriptive studies on consumer behavior [37,38]. Study participants met the following inclusion criteria: (a) being of legal age, (b) being Peruvian, (c) residing in Peru, and (d) having consumed Andean grains in the last 6 months.

The cultural context of origin was operationalized based on participants’ self-reported place of origin, defined as the region where they were born or spent most of their formative years, and classified into three categories: traditional Andean context (Sierra), urban-coastal context (Costa), and Amazonian context (Selva). This variable was used as a geographic and territorial proxy for cultural proximity to Andean grain production systems, based on the assumption that growing up in regions with a history of Andean grain cultivation is associated with greater cultural familiarity and habitual exposure to these products. This study did not directly measure cultural identity as a psychological construct; therefore, the cultural context of origin should be interpreted as a sociodemographic indicator of territorial origin rather than a measure of cultural self-identification.

The final sample consisted of *N* = 1074 participants, exceeding the minimum thresholds recommended for descriptive studies with frequency and percentage analyses [39] and lending statistical robustness to the results. Data collection was carried out using a Google Form, in which the research purpose was explained. Before completing the survey, participants could provide informed consent and withdraw at any time. All information collected was treated anonymously and used exclusively for research purposes. In this paper, the term ‘Peruvian consumers’ refers to the profile of participants selected through convenience sampling, who are predominantly young university affiliates and therefore should not be perceived as representative of the broader category of Peruvian consumers.

The inclusion criterion of prior consumption within the preceding 6 months established a minimum threshold of familiarity with the product category, not a requirement for habitual consumption. The subsequent frequency questions asked about typical behavioral patterns without a fixed reference period. Therefore, participants who had consumed Andean grains on a single occasion within the six-month window may have reported “Never” or “Almost never” on the frequency scales, as these responses reflect their general consumption habits rather than contradicting the inclusion criterion.

### 3.2. Measures

A self-administered survey was used, which included eight sections: (a) types of Andean grains usually consumed (multiple choice), (b) usual way of consuming (single response), (c) frequency of consumption (five-level ordinal scale), (d) usual purchase channel (single response), (e) reasons for purchasing (multiple choice), (f) motivations for consumption (multiple choice), and (g) factors that hinder or limit consumption (single response). The items were developed based on the theoretical framework established in the research literature review.

The multiple-choice questions were based on the “check-all-that-apply” (CATA) method of responding, which is advised for obtaining information about the multiple aspects of food buying judgments and motives [40]. The frequency questions employed a five-point ordinal scale using descriptive anchors (never, infrequently, sometimes, often, and very often) in line with the scales used in earlier research related to healthy food consumption [41,42]. Appendix A (Table A1) presents the complete operationalization of the study variables, including the measurement type, response format, and response options for each item.

The questionnaire was developed in an ad hoc manner based on the theoretical framework established in the literature review. Before large-scale implementation, the questionnaire underwent two validation stages. In the first stage, the experts assessed the content validity. Five experts, including three academics specializing in consumer behavior and food marketing and two nutritionists knowledgeable about Andean grains, evaluated the items for relevance, clarity, and completeness using a four-point rating scale. The Content Validity Index (CVI) was calculated for the items and the overall instrument [43]. In the second stage, the questionnaire was tested on 30 Peruvian consumers who met the inclusion criteria and were not included in the final sample. During testing, consumers completed the questionnaire and identified the items they found unclear or difficult to answer.

### 3.3. Ethical Considerations and Data Collection Procedures

The survey was distributed via digital platforms in 2024. The Ethics Committee of the Graduate School of the Peruvian Union University approved the research under protocol number 2024-CE-EPG-00094 on 11 June 2024, and it was conducted in accordance with the Declaration of Helsinki [44]. The first part of the questionnaire included information about the research aims and clearly indicated that participation was voluntary, anonymous, and confidential. We obtained informed consent from all participants through the online questionnaire. To improve data quality, we applied control filters to detect inconsistent or out-of-range responses. For multiple-choice questions, we cleaned responses to identify a small number of records containing content irrelevant to the question, such as statements of no purchase or out-of-context responses. We excluded these records only from the analysis of the corresponding variable and retained them in the remaining analyses. Consequently, the valid sample size (*N*) may differ slightly from the total sample size (*N*) (1074) depending on the variable analyzed; we report this difference in the footnote of each corresponding table.

### 3.4. Data Analysis

The data analysis was quantitative, with both descriptive and inferential components. In the first phase, we calculated absolute (*n*) and relative (%) frequencies for all study variables to characterize the sample’s purchasing and consumption patterns, motivations, and barriers. For the multiple-choice questions in CATA format, types of Andean grains consumed, reasons for purchase, and consumption motivations, the percentages were calculated based on the valid *N* for each dimension, since each respondent could select more than one option simultaneously, and therefore, the relative frequencies did not sum to 100% [40]. The total number of mentions and the average number of responses per respondent are reported for each dimension. For the single-response variables, usual presentation, purchase frequency, consumption frequency, purchase channel, and main barrier, the percentages were calculated based on the valid *N* for each variable and totaled 100%. We further analyzed ordinal frequency variables using cumulative percentages to identify high- and low-frequency segments. A small number of records with content not relevant to the question asked were excluded only from the analysis of the corresponding variable, remaining in the rest of the analysis; consequently, the valid *N* may differ slightly from the total *N* (1074) depending on the variable analyzed, a difference that is explicitly reported in the footnote of each affected table.

In a second phase, Pearson’s chi-square tests of independence (χ^2^) were conducted to examine the associations between the three sociodemographic variables (cultural background, age group, and gender) and the four behavioral variables (purchase frequency, usual purchasing channel, main barrier to consumption, and frequency of consumption), resulting in a total of 12 cross-tabulations. The chi-squared test assesses whether the distribution of one categorical variable differs significantly across the levels of another categorical variable, making it appropriate for the nominal and ordinal scales used in this study [37,45]. The effect size of each association was quantified using Cramer’s V coefficient, a statistic that ranges from 0 to 1, is independent of sample size, and is interpreted as the effect size, with effects classified as small (V < 0.10), moderate (0.10 ≤ V < 0.30), and large (V ≥ 0.30) [46]. A significance level of α = 0.05 was established for all hypothesis tests. Statistical processing was performed using the Python programming language (version 3.12), with the scipystats module used to calculate inferential statistics [47].

## 4. Results

### 4.1. Sociodemographic Profile of the Sample

The sample comprised 1074 Peruvian consumers. Women accounted for 56.1% of respondents, and the majority were young, with 81.2% aged 25 or younger. Regarding cultural context of origin, 56.6% came from an urban-coastal environment, 36.4% from the traditional Andean context, and 7.0% from the Amazonian context (Table 1).

### 4.2. Andean Grain Purchasing and Consumption Patterns

Table 2 presents Andean grain purchase and consumption patterns. Purchase and consumption were predominantly occasional (47.0% and 42.6%, respectively), with less than one-third of respondents reporting high purchase frequency (27.9%) or high consumption frequency (32.4%). The dominant consumption presentation was raw grain for home cooking (47.3%), followed by processed products (42.2%). Local markets were the primary purchase channel (36.4%), and proximity channels (local markets and nearby stores combined) accounted for 58.7% of purchase preferences.

### 4.3. Grain Types, Purchase, Reasons, and Consumption Motivations

Table 3 presents results for the three multiple-choice dimensions. Quinoa was the most consumed grain by far (79.3%), followed by kiwicha (47.0%), cañihua (31.1%), and tar-wi (7.9%), with an average of 1.65 grain types per respondent. Regarding purchase reasons (1874 mentions; mean 1.76 per respondent), being nutritious (63.6%) and being natural (46.2%) were the leading drivers. Consumption motivations (1960 mentions; mean 1.83 per respondent) were similarly dominated by nutritional value (56.9%) and health (56.7%), with cultural identity ranking third (21.8%).

### 4.4. Barriers to the Consumption of Andean Grains

Table 4 shows the distribution of consumption barriers. High price was the most frequently cited barrier (30.7%), followed by lack of preparation knowledge (26.9%) and time scarcity (21.4%). Together, these three barriers accounted for 79.0% of all responses. Taste aversion was the least cited barrier (7.9%).

### 4.5. Associations Between Sociodemographic Variables and Consumer Behavior

Table 5 summarizes the twelve chi-square tests conducted. Nine of the twelve associations were statistically significant (*p* < 0.05), with effect sizes ranging from small to moderate. Cultural context of origin showed significant associations with all four behavioral variables (V = 0.099–0.169), making it the sociodemographic variable with the most consistent and significant associations. Age group showed significant associations with all four behavioral variables, though with smaller effect sizes than cultural context of origin, whereas sex showed a significant association only with purchase frequency.

#### 4.5.1. Cultural Context of Origin

**Figure 1:**
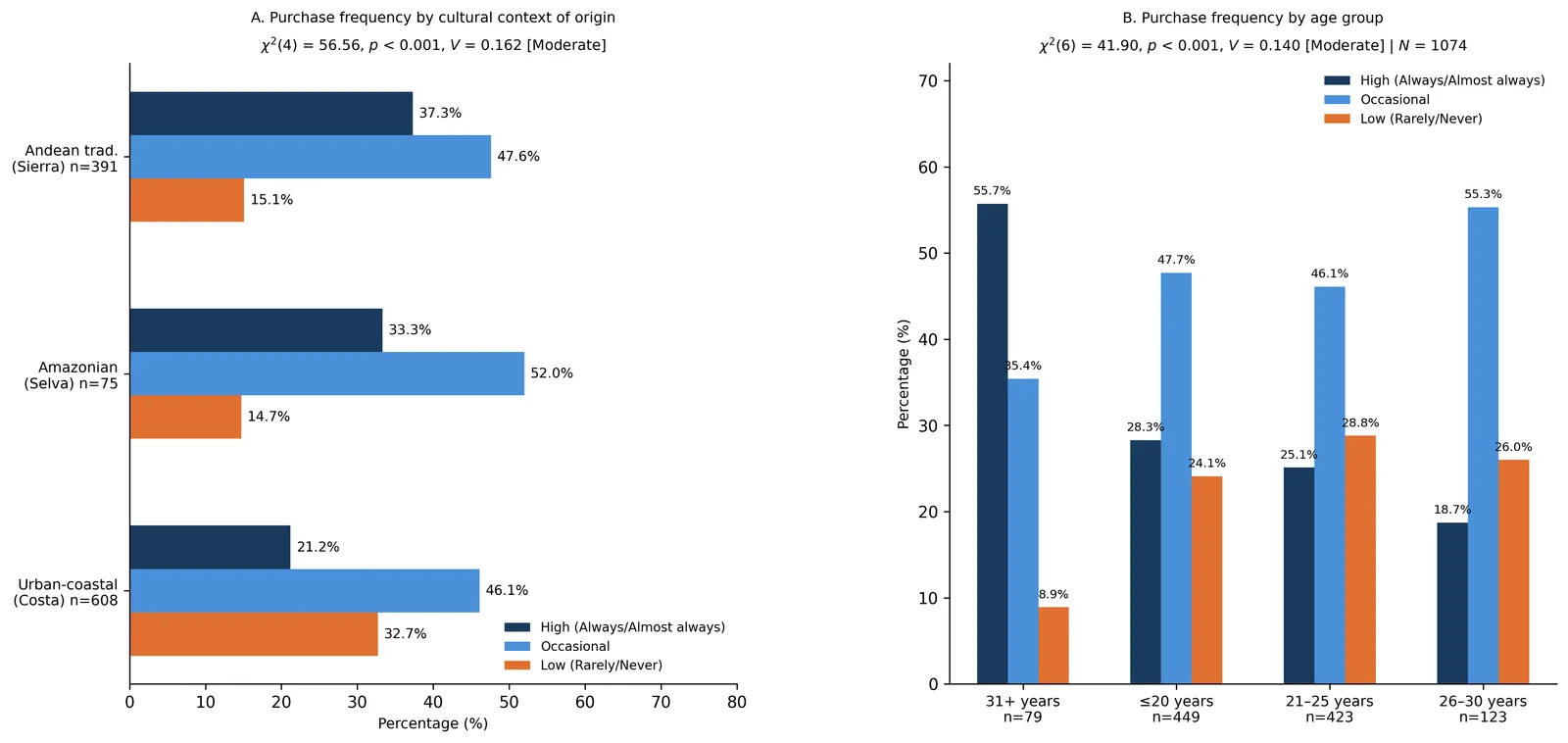
Purchase frequency of Andean grain by cultural context age group (*N* = 1074 Peruvian consumers).

**Figure 2:**
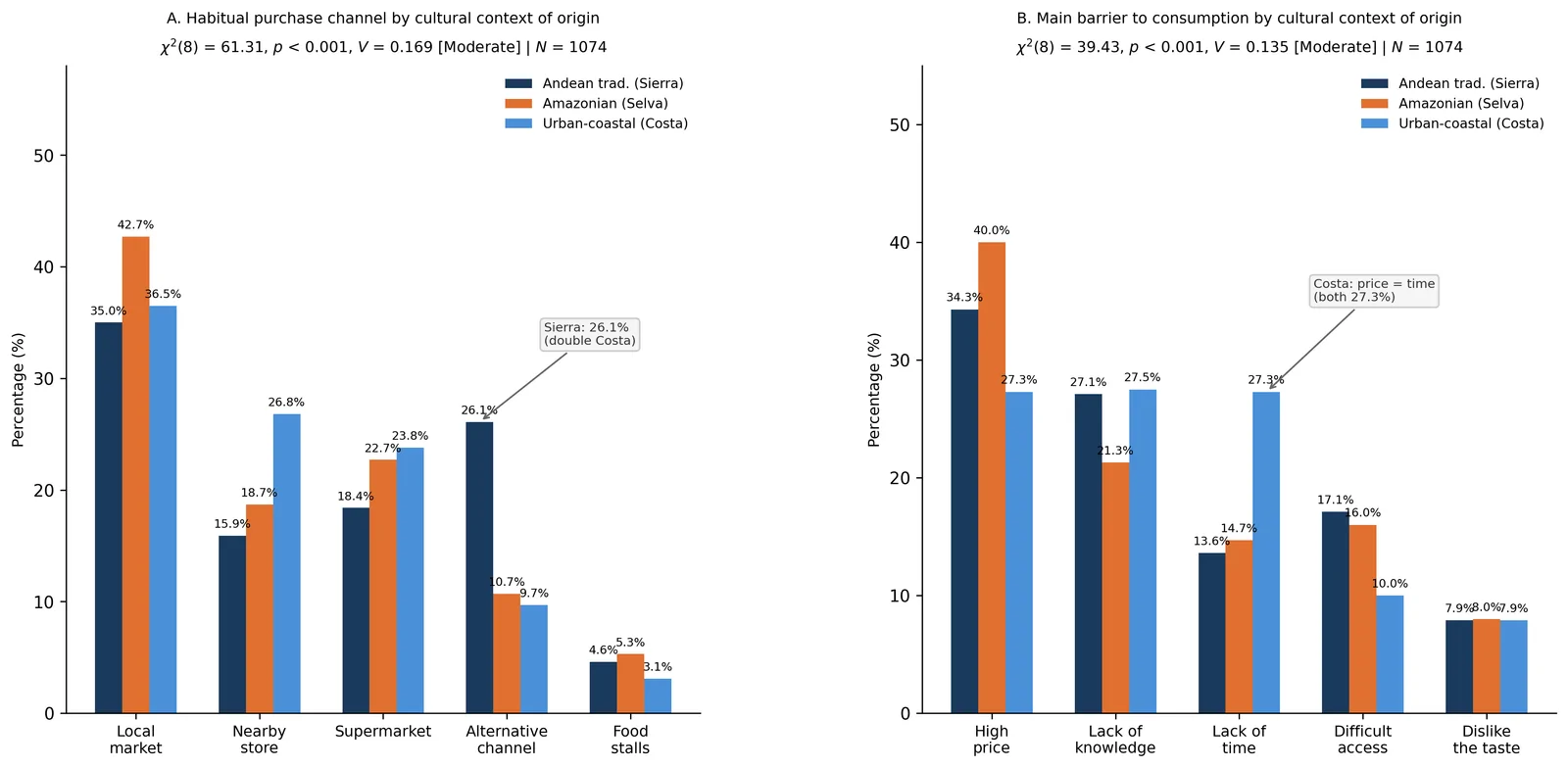
Purchase channel and main consumption barrier by cultural context of origin (*N* = 1074 Peruvian consumers).

A significant moderate association was found between cultural context and purchase frequency (χ^2^(4) = 56.56, *p* < 0.001, V = 0.162). Consumers in the traditional Andean context showed the highest proportion of high-frequency purchases (37.3%), compared with 33.3% in the Amazonian context and 21.2% in the urban-coastal context (Figure 1A). Regarding purchase channel (χ^2^(8) = 61.31, *p* < 0.001, V = 0.169), traditional Andean context consumers showed notably higher use of alternative channels (26.1%) compared to the urban-coastal context (9.7%), while coastal consumers relied more on nearby stores (26.8%) and supermarkets (23.8%) (Figure 2A). Regarding the main consumption barrier (χ^2^(8) = 39.43, *p* < 0.001, V = 0.135), high price was the dominant barrier in the Amazonian (40.0%) and Andean (34.3%) contexts, while in the urban-coastal context, time scarcity equaled price as the leading barrier (both 27.3%) (Figure 2B). A significant but small association was found with consumption frequency (χ^2^(4) = 20.92, *p* < 0.001, V = 0.099), with the Andean context recording the highest proportion of frequent consumption (40.5%) compared to 26.9% in the urban-coastal context.

#### 4.5.2. Age Group

A significant moderate association was found between age group and purchase frequency (χ^2^(6) = 41.90, *p* < 0.001, V = 0.140). Consumers aged 31 years or older showed the highest proportion of high-frequency purchases (55.7%), while the 21–25 and 26–30 age groups had the highest proportions of low-frequency purchases (28.8% and 26.0%, respectively) (Figure 1B). A significant moderate association was also found with consumption frequency (χ^2^(6) = 22.14, *p* < 0.01, V = 0.102).

#### 4.5.3. Sex

Women showed a significantly higher proportion of high-frequency purchase than men (32.1% vs. 22.6%; χ^2^(2) = 13.04, *p* < 0.01, V = 0.110). No significant associations were found between sex and purchase channel (χ^2^(4) = 2.69, ns), main consumption barrier (χ^2^(4) = 3.17, ns), or consumption frequency (χ^2^(2) = 4.79, ns).

## 5. Discussion

This study characterized purchase and consumption patterns, motivations, and barriers of 1074 Peruvian consumers regarding Andean grains, and examined their associations with sociodemographic variables, with four specific objectives.

The sample was predominantly young consumers (81.2% aged ≤25 years; 92.4% aged ≤30 years), so the findings are particularly descriptive of this age segment’s behavioral patterns. The predominantly occasional purchase and consumption pattern observed may partly reflect the economic and lifestyle constraints characteristic of young adults and university students, rather than a general indifference toward these products across the broader population.

Andean grain purchases and consumption are predominantly occasional: only 27.9% of respondents reported high purchase frequency, and only 32.4% consume them at least once a week. This pattern is consistent with the gap between positive perception and habitual behavior documented in the literature on healthy foods [13,20,21]. The relationship between positive motivations and reduced product purchase and consumption cannot be established directly, though it can be inferred indirectly by comparing the high levels of positive motivations reported for purchasing with the low levels of consumption engagement.

The higher amount of quinoa (79.3%) compared to cañihua (31.1%) and tarwi (7.9%) may be related to differences in market exposure and consumer familiarity, rather than to differences in the nutritional values of the foods themselves [31,32]. This is supported by results from other studies showing that the level of consumption of traditional foods can be explained by consumer familiarity rather than by the foods’ properties [25]. The prevalence of raw grain for home cooking (47.3%) reflects a sustained connection with traditional culinary practices, though it also introduces cognitive and time constraints regarding the regularity of consumption [29]. The large share of purchases made at local stores by consumers (58.7% in total) indicates the persistence of traditional food distribution schemes in Peru, even amid the modernization of retail trade in Latin America [35,36]. This finding has direct strategic implications: distribution strategies for Andean grains must prioritize traditional channels as a primary access point rather than relying exclusively on supermarket shelf presence. At the same time, the 15.7% share of consumers using alternative channels, organic fairs and direct-from-producer sales indicates a segment of consumers with greater awareness of product origin, suggesting an opportunity for short supply chains that connect producers directly with this motivated consumer segment [48].

A health orientation dominates both purchase reasons and consumption motivations: nutritional value (63.6%) and the natural character of the product (46.2%) led the purchase reasons, while “they are nutritious” (56.9%) and “they are healthy” (56.7%) were cited with virtually the same frequency as main motivations. This motivational profile is consistent with the multidimensional framework of food choice proposed by Steptoe et al. [27] and with recent evidence that identifies health awareness as a central antecedent of the intention to purchase healthy foods among Peruvian consumers [23,24].

Cultural identity as a consumption motivation (21.8%) indicates that Andean grains hold a symbolic and heritage dimension for a significant portion of respondents, extending beyond their nutritional value. This finding is particularly noteworthy given that 56.6% of the sample comes from an urban-coastal context, where cultural connections to Andean agricultural systems are expected to be weaker. The presence of cultural identity as a motivation even in this segment may suggest that the patrimonial value of Andean grains could extend beyond the geographical boundaries of their production regions [25,26]. This finding aligns with studies indicating that self-identity and the moral obligation to consume traditional products have been found to be significantly associated with purchase intention for Andean grains in prior studies [6,25]. Although the present study did not directly measure these constructs, such mechanisms can only be considered as possible explanations consistent with the observed pattern. The coexistence of functional, symbolic, and habitual motivations (custom: 9.5%) confirms the multidimensional nature of Andean grain consumption and suggests that promotional strategies focused exclusively on nutritional attributes would not capture the motivational factors relevant to all consumer segments [21].

High price (30.7%), lack of preparation knowledge (26.9%), and lack of time (21.4%) collectively accounted for 79.0% of the reported barriers. These three factors are consistent with the dimensions that the literature identifies as the main limitations of perceived behavioral control in healthy food consumption [22,49]; although perceived behavioral control was not directly measured in this study, this alignment should be considered a possible interpretive framework rather than an empirically demonstrated relationship.

The economic barrier is especially significant in the Peruvian context, where the quinoa export boom has driven up domestic prices, creating a paradox between abundant production and limited local accessibility [15,33,50]. This finding aligns with broader evidence that economic access to healthy diets remains limited across Latin America [51], although the present sample did not directly assess income levels or food insecurity. The cognitive barrier, a lack of knowledge about preparation methods, acts both as an inhibitor of current consumption and as an obstacle to the future incorporation of new consumers, especially in the case of cañihua and tarwi, whose preparation processes are less well-known among the urban population [29,52]. Taste rejection was the least frequent barrier (7.9%), suggesting that sensory characteristics are not a primary obstacle for most respondents in this sample, though organoleptic acceptability was not formally assessed.

The cultural background of origin showed the strongest associations, with moderate statistical relationships with the four behavioral variables analyzed (V = 0.099–0.169, all *p* < 0.001). Consumers from the traditional Andean context exhibited the highest proportion of frequent purchases (37.3% vs. 21.2% in the urban-coastal context), greater use of alternative distribution channels (26.1% vs. 9.7%), and a barrier profile dominated by price. Consumers from the urban-coastal context showed less frequent purchases and reported lack of time as the main barrier, as well as price (both 27.3%), which is consistent with the time constraints typical of metropolitan lifestyles documented in Peruvian quinoa consumers [51,53].

These results indicate that cultural proximity to the Andean production environment is associated with more regular purchasing and consumption of Andean grains. The mechanisms underlying this association, whether related to cultural familiarity, habitual exposure, social norms, or access to distribution, could not be determined from the present data and represent productive directions for future explanatory research. The cultural context of origin was operationalized as a geographic proxy variable based on self-reported place of origin; therefore, it is interpreted as reflecting territorial differences in food exposure and culinary traditions, rather than evidence of cultural identity influences. This association is consistent with what has been proposed in the literature regarding the role of cultural identity and social norms in the consumption of traditional foods [25]. Although the present study did not directly measure these constructs or perform a formal statistical moderation analysis. Future explanatory studies could test whether such mechanisms mediate the associations observed here.

Age group showed a significant moderate effect with purchase frequency (χ^2^(6) = 41.90, *p* < 0.001, V = 0.140): consumers aged 31 and over reported the highest purchase regularity (55.7%), while the 21–30 age group showed the highest proportions of low frequency, which may be partly related to the economic and lifestyle constraints typical of the university life stage [53,54], although income and lifestyle factors were not directly measured in this study. This pattern is consistent with evidence that food purchasing habits tend to become more established with age [55,56]. Women showed a higher purchase frequency than men (V = 0.110, *p* < 0.01), consistent with evidence of gender differences in health-oriented purchasing [57], though sex was not significantly associated with consumption frequency, channel, or barriers.

### 5.1. Theoretical and Practical Implications

This study makes three contributions to the literature on consumer behavior regarding food. First, it provides descriptive evidence consistent with the gap between positive valuation and habitual behavior in the context of Andean grain consumption in Peru and extends the documentation of this phenomenon to a food category with deep cultural roots in Latin America [20,21]. Second, it shows that cultural background is statistically associated with differences in purchase frequency, distribution channel, and barrier profile; these associations are consistent with, though do not directly test, constructs proposed in the literature such as subjective norms and perceived behavioral control [6,58]. Third, it provides empirical evidence on the coexistence of functional (health, nutritional value), symbolic (cultural identity), and habitual (custom) motivations among Peruvian consumers of Andean grains, thus contributing to the development of multidimensional frameworks for food choice [21,27].

For policymakers, the concentration of 79% of barriers in three actionable dimensions, namely price, knowledge, and time, provides a clear framework for intervention priorities. Strategies for the traditional Andean context should include mechanisms to reduce prices (such as public procurement, subsidies, and community feeding programs), while strategies for urban and coastal contexts should focus on culinary education and simplifying cooking techniques. For food industry actors, the dual dominance of health and cultural identity motivations suggests a communication strategy that integrates nutritional messaging with heritage and cultural narratives, without changes in taste, since the sensory barrier effect is minimal. The primacy of traditional channels suggests that distribution strategies should prioritize local markets alongside modern retail channels.

### 5.2. Limitations and Future Research

This study has several limitations. Convenience sampling does not allow us to generalize our findings to the entire Peruvian population. This study uses a cross-sectional design, thus precluding the authors from inferring causal relationships among the variables under consideration. The authors focused primarily on young participants (81.2% were 25 years old or younger). Future studies should use probabilistic sampling strategies to ensure adequate representation across age groups, geographic regions, and socioeconomic levels. Finally, because the study relies on self-reported behaviors, social desirability bias may be present.

These limitations suggest four productive directions for future research. First, researchers can use an explanatory approach, along with structural equation modeling (SEM or PLS-SEM), to investigate whether constructs such as cultural identity, health consciousness, and perceived behavioral control mediate or moderate the relationship between sociodemographic factors and consumer behavior regarding Andean grains. Second, implementing longitudinal research designs will allow researchers to evaluate the transition from occasional consumption to regular consumption and understand how it happens. Third, segmentation studies may help market researchers use cluster analysis or latent class analysis to identify specific consumer segments and develop targeted marketing strategies. Fourth, discrete choice experiments or contingent valuation studies may help assess how much consumers are willing to pay for Andean grains with additional value, such as organic certification, place of origin, and sustainable production.

Additionally, the inclusion criterion and the frequency questions operated with different reference periods: while participants were required to have consumed Andean grains in the preceding 6 months, the frequency scales measured habitual behavioral patterns without a fixed time frame. This difference in reference periods explains the small proportion of respondents who reported “Never” on the frequency variables despite meeting the inclusion criterion, and future studies should address this discrepancy by aligning the reference periods of both measures.

## 6. Conclusions

This study analyzes consumer behavior regarding Andean grains among a predominantly young Peruvian sample, in which 81.2% of respondents were 25 years old or younger, characterizing purchasing and consumption patterns, as well as consumer motivations and barriers, by cultural background, age group, and gender.

Quinoa dominates consumption (79.3%), while cañihua and tarwi, two grains underutilized despite their nutritional quality, show disparities in commercial visibility and consumer familiarity rather than differences in nutritional quality. Purchases and consumption were sporadic, indicating a gap between positive perceptions and regular access in consumers’ consumption patterns, a gap that awareness campaigns cannot bridge.

Health-oriented factors, represented by nutritional value and naturalness as the most important purchasing factors, and by nutritional and health benefits as the primary motivations, were the main drivers of Andean grain consumption. Cultural identity was an important secondary motivation, indicating that these products have a symbolic or heritage dimension in addition to their functional attributes.

High price, limited knowledge of how to prepare the product, and lack of time together accounted for 79.0% of the barriers identified. The low prevalence of taste aversion among the surveyed consumer group shifts intervention priorities toward affordability, culinary education, and practical cooking methods rather than product reformulation.

The cultural context of origin showed the strongest sociodemographic links to consumer behavior: consumers from the traditional Andean context purchased these products more regularly, used alternative channels more frequently, and reported predominantly economic barriers, whereas consumers from coastal urban areas reported a mixed profile of economic and time-related barriers. Effective interventions should vary according to cultural and territorial context. Peru, the world’s leading producer of Andean grains, has the productive base needed for these crops to become a pillar of the national diet; this study provides an empirical foundation for producers, public policymakers, and educators working toward this goal.

## Figures and Tables

**Table 1:** Demographic information (*N* = 1074).

Variable	Category	*n*	%
Gender	Female	601	56.00
	Male	473	44.00
Age group	18–20 years old	449	41.80
	21–25 years old	423	39.40
	26–30 years old	123	11.50
	31+ years	79	7.40
Cultural context	Urban-coastal context (Coast)	608	56.60
of origin	Traditional Andean context (Sierra)	391	36.40
	Amazonian context (Jungle)	75	7.00

**Table 2:** Purchase and consumption patterns of Andean grains (*N* = 1074).

Variable	Category	*n*	%
Purchase frequency	Always/Almost always	300	27.9
	Occasionally	505	47.0
	Almost never/Never/Doesn’t know	269	25.1
Frequency of consumption	High (≥1 time/week)	348	32.4
	Occasional (1–3 times/month)	458	42.6
	Low (rarely/never)	268	25.0
Typical consumer presentation	Raw grain for cooking (at home)	505	47.3
	In processed products (cookies, cereals, bars, etc.)	451	42.2
	In meals prepared outside the home	112	10.5
Usual purchasing channel	Local market	391	36.4
	Store near home	239	22.3
	Supermarket	234	21.8
	Organic markets/Direct from the producer/Online	169	15.7
	Food stalls	41	3.8

Note: Purchase and consumption frequency categories were grouped into three levels for ease of reading: high = always + almost always (purchase) or ≥1 time/week (consumption); low = almost never + never + don’t know (purchase) or rarely + never (consumption). The category ‘Organic Fairs/Direct Purchase from Producer/Online’ groups alternative channels with lower frequency.

**Table 3:** Types of grains consumed, reasons for purchase, and consumption motivations.

Variable/Dimension	Category	Mentions (*n*)	% of *N*
Andean grains consumed (*N* = 1074)	Quinoa	852	79.3
	Kiwicha	505	47.0
	Cañihua	334	31.1
	Tarwi	85	7.9
Reasons for purchase (valid *N* = 1065)	Because they are nutritious	677	63.6
	Because they are natural	492	46.2
	Because they taste good	303	28.5
	Because they have a good price/quality ratio	173	16.2
	Because it was recommended to me	148	13.9
	Because they are easy to find	81	7.6
Consumption motivations (valid *N* = 1071)	They are nutritious	609	56.9
	They are healthy	607	56.7
	They are part of my culture	234	21.8
	I like its flavor	226	21.1
	They are accessible	182	17.0
	Out of habit	102	9.5

Note: Multiple-choice question (check all that apply, CATA): percentages are calculated based on the *N* for each dimension and do not sum to 100%. For purchase reasons, 9 invalid responses are excluded (valid *N* = 1065; average 1.76 reasons/respondent). For consumption motivations, 3 invalid responses are excluded (valid *N* = 1071; average 1.83 motivations/respondent).

**Table 4:** Factors that hinder or limit the consumption of Andean grains (*N* = 1074).

Barrier to Consumption	*n*	%	Type of Barrier
High price	330	30.7	Economic
Lack of knowledge about how to prepare them	289	26.9	Cognitive
Lack of time for preparation	230	21.4	Temporary
Difficult access to the product	140	13.0	Logistics
Dislike of the taste	85	7.9	Sensory
Total	1074	100.0	

Note: Single categorized response. The three main barriers (price, lack of knowledge, and lack of time) account for 79.0% of the mentions. The classification by barrier type was developed by the authors based on the nature of each identified factor: economic (high price), cognitive (lack of knowledge about preparation), temporal (lack of available time), logistical (difficulty accessing the product), and sensory (taste rejection), following the distinction between individual and structural barriers proposed in the literature on eating behavior [21,42].

**Table 5:** Chi-square tests: sociodemographic variables × behavior.

Dependent Variable	Cultural Context of Origin (*n* = 1074)		Age Group (*n* = 1074)		Gender (*n* = 1074)		Main Interpretation (f)
	χ^2^ (df)	V	χ^2^ (df)	V	χ^2^ (df)	V	
Purchase frequency	56.6 (4)	0.162 ***	41.9 (6)	0.140 ***	13.0 (2)	0.110 **	Highlands and jungle > coast (high purchase); women > men; 31+ years > rest.
Purchase channel	61.3 (8)	0.169 ***	23.2 (12)	0.085 *	2.7 (4)	0.050 ns	Sierra uses the alternative channel more (26.1%). Costa: nearby store and supermarket.
Barrier to consumption	39.4 (8)	0.135 ***	29.8 (12)	0.096 **	3.2 (4)	0.054 ns	Price dominates in Sierra/Selva. Time = price in Costa; ns for gender.
Frequency of consumption	20.9 (4)	0.099 ***	22.1 (6)	0.102 **	4.8 (2)	0.067 ns	Sierra: higher frequency of consumption (40.5%). Moderate age effect. Not applicable for gender.

Note. df = degrees of freedom. V = Cramér’s V (effect size: small, < 0.10; moderate, 0.10–0.29; large, ≥ 0.30; [46]). Significance level is indicated by asterisks next to V: *** *p* < 0.001, ** *p* < 0.01, * *p* < 0.05, and ns = not significant. ‘Main interpretation’ summarizes the direction of the largest percentage of differences between categories.

## Data Availability

The original contributions presented in this study are included in the article. Further inquiries can be directed to the corresponding author.

## Appendix A

Table A1 presents the complete operationalization of the eight variables measured in this study, including each item’s variable type, response format, and response options.

**Table A1:** Operationalization of study variables.

Variable	Type	Response Format	Response Options
Types of Andean grains consumed	Behavioral	CATA (multiple choice)	Quinoa, kiwicha, Cañihua, Tarwi
Usual consumption form	Behavioral	Single response	Raw grain (at home), processed products, meals eaten out
Purchase frequency	Behavioral	5-level ordinal scale	Never, almost never, occasionally, almost always, always
Consumption frequency	Behavioral	5-level ordinal scale	Never, rarely, occasionally, frequently, very frequently
Usual purchase channel	Behavioral	Single response	Local market, nearby store, supermarket, alternative channel, food stalls
Reasons for purchase	Attitudinal-cognitive	CATA (multiple choice)	Nutritious, natural, good taste, good value, recommendation, easy to find
Consumption motivations	Attitudinal-affective	CATA (multiple choice)	Nutritious, healthy, cultural identity, flavor, affordable, custom
Barriers to consumption	Perceptual	Single response	High price, lack of knowledge, lack of time, difficult to access, dislikes the taste
Cultural context of origin	Sociodemographic, territorial indicator	Single answer	Coast (urban-coastal); highlands (traditional Andean); jungle (Amazonian)

Note that CATA means check all that apply. For CATA queries, respondents could select multiple answers simultaneously; therefore, relative frequencies should not add up to 100% but should be calculated over the valid N for each dimension. The scales of measuring frequencies on 5 levels are similar to the scales that were utilized in previous studies related to healthy food habits and behaviors [41,42]. Responses or alternatives for each variable were developed based on the theoretical foundation discussed in Section 2, but adjusted to the regional peculiarities of Andean grains in Peru.

## References

[1] Bazile D. (2021). Quinoa, A Model Crop for Tomorrow’s Agriculture. Biology and Biotechnology of Quinoa.

[2] Prajapati A., Singh A.K. (2024). Quinoa: Nutrition Profile, Processing and Food Products. Pseudocereals.

[3] Choque Delgado G.T., Carlos Tapia K.V., Pacco Huamani M.C., Hamaker B.R. (2023). Peruvian Andean Grains: Nutritional, Functional Properties and Industrial Uses. Crit. Rev. Food Sci. Nutr..

[4] Guerrero Ardiles F.D., Bohórquez-Medina S.L., Bohórquez-Medina A.L. (2025). Andean Food Consumption and Perceived Physical Performance in University Athletes. Nutr. Clínica Dietética Hosp..

[5] Tridge Peru Leads the Production and Export of Quinoa. https://www.tridge.com/market-overview/quinoa.

[6] Millones-Liza D.Y., García-Salirrosas E.E., Mayta-Pinto E. (2025). Factors That Influence the Purchase Intention of Andean Grains in University Students in the Peruvian Market. Foods.

[7] Cheng L., Cui H., Zhang Z., Yang M., Zhou Y. (2024). Study on Consumers’ Motivation to Buy Green Food Based on Meta-Analysis. Front. Sustain. Food Syst..

[8] Martinho V.J.P.D., Bartkiene E., Djekic I., Tarcea M., Barić I.C., Černelič-Bizjak M., Szűcs V., Sarcona A., El-Kenawy A., Ferreira V. (2022). Determinants of Economic Motivations for Food Choice: Insights for the Understanding of Consumer Behaviour. Int. J. Food Sci. Nutr..

[9] Vidgen H.A. (2025). Determinants of Food Choice Helen A. Vidgen. Food and Nutrition.

[10] Rodríguez-Guerra V., Sanz-Cañada J., Belletti G., Marescotti A., Mengoni M. (2026). Food-Related Values and Consumption Barriers in Alternative Food Networks in Central-Western Tuscany and the Region of Madrid. Sustainability.

[11] Chung S.-J., Kim M.-R. (2026). Understanding International Food Consumers by Preferences, Values and Attitudes Across Cultures. Food and Consumer Behavior: A Comprehensive Reference.

[12] García-Salirrosas E.E., Haro-Zea K.L., Acevedo-Duque Á., Urraca-Vergara E.M., Flores Rivera A.R., Millones-Liza D.Y. (2026). Explaining Willingness to Pay for Andean Grains Through Health Consciousness. Foods.

[13] Vermeir I., Verbeke W. (2006). Sustainable Food Consumption: Exploring the Consumer “Attitude—Behavioral Intention” Gap. J. Agric. Environ. Ethics.

[14] Bazile D., Jacobsen S.E., Verniau A. (2016). The Global Expansion of Quinoa: Trends and Limits. Front. Plant Sci..

[15] Jacobsen S.-E. (2011). The Situation for Quinoa and Its Production in Southern Bolivia: From Economic Success to Environmental Disaster. J. Agron. Crop Sci..

[16] Vega-Gálvez A., Miranda M., Vergara J., Uribe E., Puente L., Martínez E.A. (2010). Nutrition Facts and Functional Potential of Quinoa (*Chenopodium quinoa* Willd.), an Ancient Andean Grain: A Review. J. Sci. Food Agric..

[17] Repo-Carrasco R., Espinoza C., Jacobsen S.-E. (2003). Nutritional Value and Use of the Andean Crops Quinoa (*Chenopodium quinoa*) and Kañiwa (*Chenopodium pallidicaule*). Food Rev. Int..

[18] Kotler P., Keller K.L., Chernev A. (2021). Marketing Management.

[19] Solomon M.R. (2020). Consumer Behavior: Buying, Having, and Being.

[20] Verbeke W. (2005). Consumer Acceptance of Functional Foods: Socio-Demographic, Cognitive and Attitudinal Determinants. Food Qual. Prefer..

[21] Bourke B.C., McCarthy M.B., McCarthy S.N. (2024). Behavioural Factors Influencing Consumer Acceptance of Sustainable Healthy Food: A Review and Research Agenda. Int. J. Consum. Stud..

[22] Baker M.T., Lu P., Parrella J.A., Leggette H.R. (2022). Consumer Acceptance toward Functional Foods: A Scoping Review. Int. J. Environ. Res. Public Health.

[23] García-Salirrosas E.E., Escobar-Farfán M., Esponda-Perez J.A., Villar-Guevara M., Rondon-Eusebio R.F., Ezcurra-Zavaleta G., Urraca-Vergara E.M., Guerra-Velásquez M. (2025). Healthy Lifestyle Motivators of Willingness to Consume Healthy Food Brands: An Integrative Model. Foods.

[24] Müller-Pérez J., Acevedo-Duque Á., García-Salirrosas E.E., Escobar-Farfán M., Esponda-Pérez J.A., Cachicatari-Vargas E., Álvarez-Becerra R., Alcina De Fortoul S. (2025). Factors Influencing Healthy Product Consumer Behavior: An Integrated Model of Purchase Intention. Front. Public Health.

[25] Guerrero L., Guàrdia M.D., Xicola J., Verbeke W., Vanhonacker F., Zakowska-Biemans S., Sajdakowska M., Sulmont-Rossé C., Issanchou S., Contel M. (2009). Consumer-Driven Definition of Traditional Food Products and Innovation in Traditional Foods. A Qualitative Cross-Cultural Study. Appetite.

[26] Almli V.L., Verbeke W., Vanhonacker F., Næs T., Hersleth M. (2011). General Image and Attribute Perceptions of Traditional Food in Six European Countries. Food Qual. Prefer..

[27] Steptoe A., Pollard T.M., Wardle J. (1995). Development of a Measure of the Motives Underlying the Selection of Food: The Food Choice Questionnaire. Appetite.

[28] Darmon N., Drewnowski A. (2008). Does Social Class Predict Diet Quality?. Am. J. Clin. Nutr..

[29] Carvajal-Larenas F.E., Linnemann A.R., Nout M.J.R., Koziol M., van Boekel M.A.J.S. (2016). Lupinus Mutabilis: Composition, Uses, Toxicology, and Debittering. Crit. Rev. Food Sci. Nutr..

[30] Jabs J., Devine C.M. (2006). Time Scarcity and Food Choices: An Overview. Appetite.

[31] Villacrés E., Quelal M., Galarza S., Iza D., Silva E. (2022). Nutritional Value and Bioactive Compounds of Leaves and Grains from Quinoa (*Chenopodium quinoa* Willd.). Plants.

[32] Gonyea A., Esteban E., Repo-Carrasco-Valencia R., Martinez M. (2025). Grains of Wisdom: Insights into the Minds of Top Chefs—A Synthesis of Expert Interviews and Literature. Int. J. Gastron. Food Sci..

[33] Alandia G., Rodriguez J.P., Jacobsen S.-E., Bazile D., Condori B. (2020). Global Expansion of Quinoa and Challenges for the Andean Region. Glob. Food Sec..

[34] MIDAGRI (2024). Innovación En La Cadena de Valor de La Quinua.

[35] Reardon T., Timmer C.P., Barrett C.B., Berdegué J. (2003). The Rise of Supermarkets in Africa, Asia, and Latin America. Am. J. Agric. Econ..

[36] McKinsey and Company (2024). The State of Grocery Retail in Latin America.

[37] Malhotra N. (2020). Marketing Research: An Applied Orientation.

[38] Saunders M., Lewis P.L., Thornhill A. (2023). Research Methods for Business Students.

[39] Hair J.F., Hult G.T.M., Ringle C.M., Sarstedt M., Castillo Apraiz J., Cepeda Carrión G.A., Roldán J.L. (2019). Manual de Partial Least Squares Structural Equation Modeling (PLS-SEM) (Segunda Edición).

[40] Ares G., Varela P. (2017). Trained vs. Consumer Panels for Analytical Testing: Fueling a Long Lasting Debate in the Field. Food Qual. Prefer..

[41] Sánchez-Bravo P., Chambers V E., Noguera-Artiaga L., Sendra E., Chambers E., Carbonell-Barrachina Á.A. (2021). Consumer Understanding of Sustainability Concept in Agricultural Products. Food Qual. Prefer..

[42] Pinho M.G.M., Mackenbach J.D., Charreire H., Oppert J.M., Bárdos H., Glonti K., Rutter H., Compernolle S., De Bourdeaudhuij I., Beulens J.W.J. (2017). Exploring the Relationship between Perceived Barriers to Healthy Eating and Dietary Behaviours in European Adults. Eur. J. Nutr..

[43] Lynn M.R. (1986). Determination and Quantification of Content Validity. Nurs. Res..

[44] World Medical Association (2013). World Medical Association Declaration of Helsinki: Ethical Principles for Medical Research Involving Human Subjects. JAMA.

[45] Field A. (2024). Discovering Statistic Using IBM SPSS Statistic.

[46] Cohen J. (1988). Statistical Power Analysis for the Behavioral Sciences.

[47] Virtanen P., Gommers R., Oliphant T.E., Haberland M., Reddy T., Cournapeau D., Burovski E., Peterson P., Weckesser W., Bright J. (2020). SciPy 1.0: Fundamental Algorithms for Scientific Computing in Python. Nat. Methods.

[48] Michaelidou N., Hassan L.M. (2008). The Role of Health Consciousness, Food Safety Concern and Ethical Identity on Attitudes and Intentions towards Organic Food. Int. J. Consum. Stud..

[49] McEachan R.R.C., Conner M., Taylor N.J., Lawton R.J. (2011). Prospective Prediction of Health-Related Behaviours with the Theory of Planned Behaviour: A Meta-Analysis. Health Psychol. Rev..

[50] Sanchez-Matos J., Vázquez-Rowe I., Kahhat R. (2024). Are Peruvians Moving toward Healthier Diets with Lower Environmental Burden? Household Consumption Trends for the Period 2008–2021. J. Ind. Ecol..

[51] FAO (2025). Panorama Regional de La Seguridad Alimentaria y La Nutrición En América Latina y El Caribe.

[52] Li X., Braakhuis A., Li Z., Roy R. (2022). How Does the University Food Environment Impact Student Dietary Behaviors? A Systematic Review. Front. Nutr..

[53] Higuchi A., Maehara R., Sánchez-Pérez L.d.l.Á. (2022). Factors Related to Quinoa Consumption in Peru during the COVID-19 Pandemic. Innovar.

[54] Ramos P. (2024). Prejudices and Barriers Which Explain Poor Diets Among Young University Students. https://www.uoc.edu/en/news/2024/prejudices-and-barriers-explain-poor-diets-among-young-university-students.

[55] Canova L., Bobbio A., Manganelli A.M. (2020). Predicting Fruit Consumption: A Multi-Group Application of the Theory of Planned Behavior. Appetite.

[56] Wood W., Neal D.T. (2009). The Habitual Consumer. J. Consum. Psychol..

[57] Feraco A., Armani A., Amoah I., Guseva E., Camajani E., Gorini S., Strollo R., Padua E., Caprio M., Lombardo M. (2024). Assessing Gender Differences in Food Preferences and Physical Activity: A Population-Based Survey. Front. Nutr..

[58] Ajzen I. (1991). The Theory of Planned Behavior. Organ. Behav. Hum. Decis. Process..

